# The Emerging Roles of Extracellular Vesicles As Communication Vehicles within the Tumor Microenvironment and Beyond

**DOI:** 10.3389/fendo.2017.00194

**Published:** 2017-08-08

**Authors:** Ryan Sullivan, Grace Maresh, Xin Zhang, Carlos Salomon, John Hooper, David Margolin, Li Li

**Affiliations:** ^1^Laboratory of Translational Cancer Research, Ochsner Clinic Foundation, New Orleans, LA, United States; ^2^Exosome Biology Laboratory, Centre for Clinical Diagnostics, University of Queensland Centre for Clinical Research, Royal Brisbane and Women’s Hospital, The University of Queensland, Brisbane, QLD, Australia; ^3^Maternal-Fetal Medicine, Department of Obstetrics and Gynecology, Ochsner Clinic Foundation, New Orleans, LA, United States; ^4^Faculty of Pharmacy, Department of Clinical Biochemistry and Immunology, University of Concepción, Concepción, Chile; ^5^Mater Research Institute-University of Queensland, Brisbane, QLD, Australia; ^6^Department of Colon and Rectal Surgery, Ochsner Clinic Foundation, New Orleans, LA, United States; ^7^Ochsner Clinical School, School of Medicine, University Queensland, New Orleans, LA, United States

**Keywords:** extracellular vesicle, exosome, microvesicle, cancer, tumor microenvironment

## Abstract

Tumors evolve in complex and dynamic microenvironments that they rely on for sustained growth, invasion, and metastasis. Within this space, tumor cells and non-malignant cells are in frequent communication. One specific mode of communication that has gained recent attention is the release of extracellular vesicles (EVs). EVs are lipid bilayer-bound vehicles that are released from the cell membrane and carry nucleic acids, proteins, and lipids to neighboring or distant cells. EVs have been demonstrated to influence a multitude of processes that aid in tumor progression including cellular proliferation, angiogenesis, migration, invasion, metastasis, immunoediting, and drug resistance. The ubiquitous involvement of EVs on cancer progression makes them very suitable targets for novel therapeutics. Furthermore, they are being studied as specific markers for cancer diagnostics, prognosis, and even as chemotherapy drug-delivery systems. This review focuses on the most recent advances in EV knowledge, some current and potential problems with their use, and some proposed solutions to consider for the future.

## Introduction

Cancer is a leading cause of morbidity and mortality worldwide. In 2015, it was responsible for 8.8 million deaths, making it the second leading cause of death globally (1). In the United States, nearly 40% of people will be diagnosed with cancer within their lifetimes, and the national expenditure for cancer patient care is estimated to reach $156 billion by the year 2020.

A great deal of progress has been made in understanding cancer development over the last century enabling a steady decline in cancer mortality over the past two decades (2). However, in general, there still remains a large gap in survival rates between localized and metastatic disease (3), indicating that advanced disease is more poorly understood and more difficult to treat. In fact, early detection still remains one of the most reliable ways to ascertain an effective treatment response (4). The high mortality of advanced disease calls for a better understanding of cancer cell biology and, in particular, the mediating factors leading to cancer progression. One such realm of cancer biology that is increasingly recognized as a key factor in tumor progression, metastasis, and chemotherapeutic drug resistance is the tumor microenvironment (TME) (5).

Tumors develop in complex and dynamic environments that aid their sustained growth, invasion, and metastasis (6). This space, called the TME, directly surrounds the tumor and consists of an array of non-malignant cells including fibroblasts, immune cells, adipose tissue, blood and lymphatic vessel networks, and signaling molecules of the extracellular matrix. Current research has demonstrated the vital role of the TME in maintaining and progressing the tumor phenotype and as a possible focus for targeted therapy (6). Complex interactions between the TME and malignant cells occur through a very sophisticated network of cellular communication. Many of these signaling pathways operate through direct cell-to-cell contact or by classical paracrine signaling loops of cytokines or growth factors with their receptors. However, more recently, extracellular vesicle (EV) shedding has emerged as another important mechanism of cellular interchange (7). EVs are lipid bilayer-bound vehicles that are released from the cell membrane and carry nucleic acids (DNA, mRNA, and miRNA), proteins, and lipids to neighboring or distant cells (8). EVs exhibit wide-ranging roles in maintaining normal cellular and biological physiology. Undoubtedly, however, the most heavily researched area of EV-associated pathology is their role in tumor development and chemotherapeutic resistance in cancer. In this review, we highlight the most current studies involving EVs and their influence on the pathogenesis of cancer.

## The TME

Cells reside in diverse microenvironments that help maintain physiological order within a tissue. During tumorigenesis, this region consists of tumor cells and an array of non-malignant cells including fibroblasts, immune cells, adipose tissue, blood and lymphatic vessel networks, and the extracellular matrix called the TME (5). In fact, non-cancerous cells can account for greater than 50% of the overall tumor mass (9). Within the TME, complex communication pathways are occurring among its inhabitants (10), allowing the tumor and the TME to independently influence one another and co-evolve. It is apparent that the TME has a vital role in maintaining and progressing the tumor phenotype and that it is a possible focus for targeted therapy (6).

A healthy local microenvironment surrounding a tumor may initially help to provide anticancer effects. This was first demonstrated in melanoma where the number of tumor-infiltrating lymphocytes was shown to correlate with better clinical outcomes (11). This concept has also been demonstrated in many other malignancies including colorectal cancer, where an active TME and signs of an immune response were associated with the absence of early metastasis and increased survival (12). In fact, the notion that local immune cells act as guards against nascent transformed cells was developed over 60 years ago (13).

However, as tumors develop, their microenvironment becomes disrupted, protection is lost, and tumor progression may be allowed to continue. The association between a disrupted microenvironment and malignancy is well established and is illustrated during the process of chronic inflammation (14) where local dysfunction acts to induce oncogenic mutations, genomic instability, and early tumor promotion and enhance angiogenesis (14). Tumors can directly disrupt and manipulate their local microenvironment by hijacking local cells and coercing them to provide varying services to help avoid the immune response or aid in other processes of tumor development by supplying cytokines, growth factors, and proteinases (15). Perhaps the best studied of these commandeered cells are the tumor-associated macrophage (TAM), the cancer-associated fibroblast (CAF), and the myeloid-derived suppressor cells (MDSCs).

The TAM is a pirated cell of the TME that provides local support to the tumor (16). In normal circumstances, macrophages act as a main defense against pathogens and a bridge between the innate and adaptive immune systems. As they become immersed in the evolving TME and are exposed to hypoxic conditions and tumor-derived factors, macrophages begin to change phenotype and functionality (17). This change may have serious implications for patient prognosis. A large array of clinical data indicate that the accumulation of TAMs within a tumor is associated with poorer outcomes (18). The production of TAMs was originally described as a phenotypic change of the M1 macrophage, which produces pro-inflammatory cytokines, to the M2 macrophage, known to produce anti-inflammatory and protumorigenic functions (19). This has been recently challenged in a mouse mammary cancer model that suggested TAMs are phenotypically and functionally different from M2 macrophages. The study further reports that TAMs more likely originate from CCR2^+^ inflammatory monocytes that depend on the notch signaling pathway for differentiation (20).

Cancer-associated fibroblasts are fibroblasts that have become activated by local factors within the TME (21). They have similar morphological properties to myofibroblasts (22) and assume the phenotype of a facilitator of tissue repair by generating and releasing growth factors and regulating inflammation and immunity (23). CAFs, unlike normal physiologically activated fibroblasts, are constitutively active and neither revert to a normal phenotype nor undergo apoptosis (24). They have been implicated in many aspects of tumor progression through varying mechanisms (25). For example, when CAFs isolated from human breast carcinoma were subcutaneously co-injected with a breast cancer cell line into an immunodeficient murine host, tumor proliferation was significantly increased in the presence of CAFs compared to normal mammary fibroblasts isolated from the same patient. Further experiments attributed this to the CAF’s ability to secrete stromal cell-derived factor-1 (SDF-1; also known as CXCL12) (26). It has also been shown that mice orthotopically co-xenografted with human pancreatic cancer cells and CAFs develop pancreatic tumors and metastases and that the CAF secretome stimulates the epithelial–mesenchymal transition (EMT) (27). CAFs also assist in metastasis by supplying transforming growth factor beta (TGF-β) to tumor cells, a multifunctional cytokine known to mediate the EMT (28), a process where cells lose epithelial markers and gain mesenchymal attributes allowing for a more mobile and migratory phenotype. TGF-β is also pro-angiogenic, and along with vascular endothelial growth factor (VEGF), platelet-derived growth factor (PDGF), fibroblast growth factor, and SDF-1 is released by CAFs to enhance endothelial cell proliferation and migration (25).

The MDSC is a more recently described cell type that becomes activated in pathological states and has potent immunosuppressive capacity (29). In some pathologies, MDSCs help protect the host organ from the harmful effects of excessive immune stimulation. For example, MDSCs were elevated in mice with antigen-induced autoimmune enterocolitis, and their presence lead to a reduction of disease symptoms (30). In cancer states, however, activation of MDSCs is an effective means of protection from immune-mediated killing (29) by inhibiting antigen-presenting dendritic cells, T and B cell proliferation, and natural killer cell cytotoxicity (31). Furthermore, MDSCs have also been implicated directly in tumor metastasis by helping to facilitate the EMT and to establish a distant premetastatic niche (PMN) (29).

## Communication *via* EVs within the TME

The complex interactions displayed between cancer cells and the TME, as mentioned above, occur through a very complicated network of cellular communication. Many of these signaling pathways operate through direct cell-to-cell contact or using classical paracrine signaling loops of cytokines or growth factors with their receptors. However, more recently, EV shedding has emerged as another important mechanism of cellular cross-talk (7).

Extracellular vesicles are lipid bilayer-bound vehicles that are released from the cell membrane and carry nucleic acids (DNA, mRNA, and miRNA), proteins, and lipids to neighboring or distant cells (8). Although EVs were first described over 30 years ago as being released from reticulocytes (32), they have gained significant attention only recently as key factors in regulating both normal cell physiology and disease states. They now have been identified in nearly all eukaryotic cells (33) and prokaryotic cells (34) and have been isolated from most bodily fluids (8).

**Figure 1:**
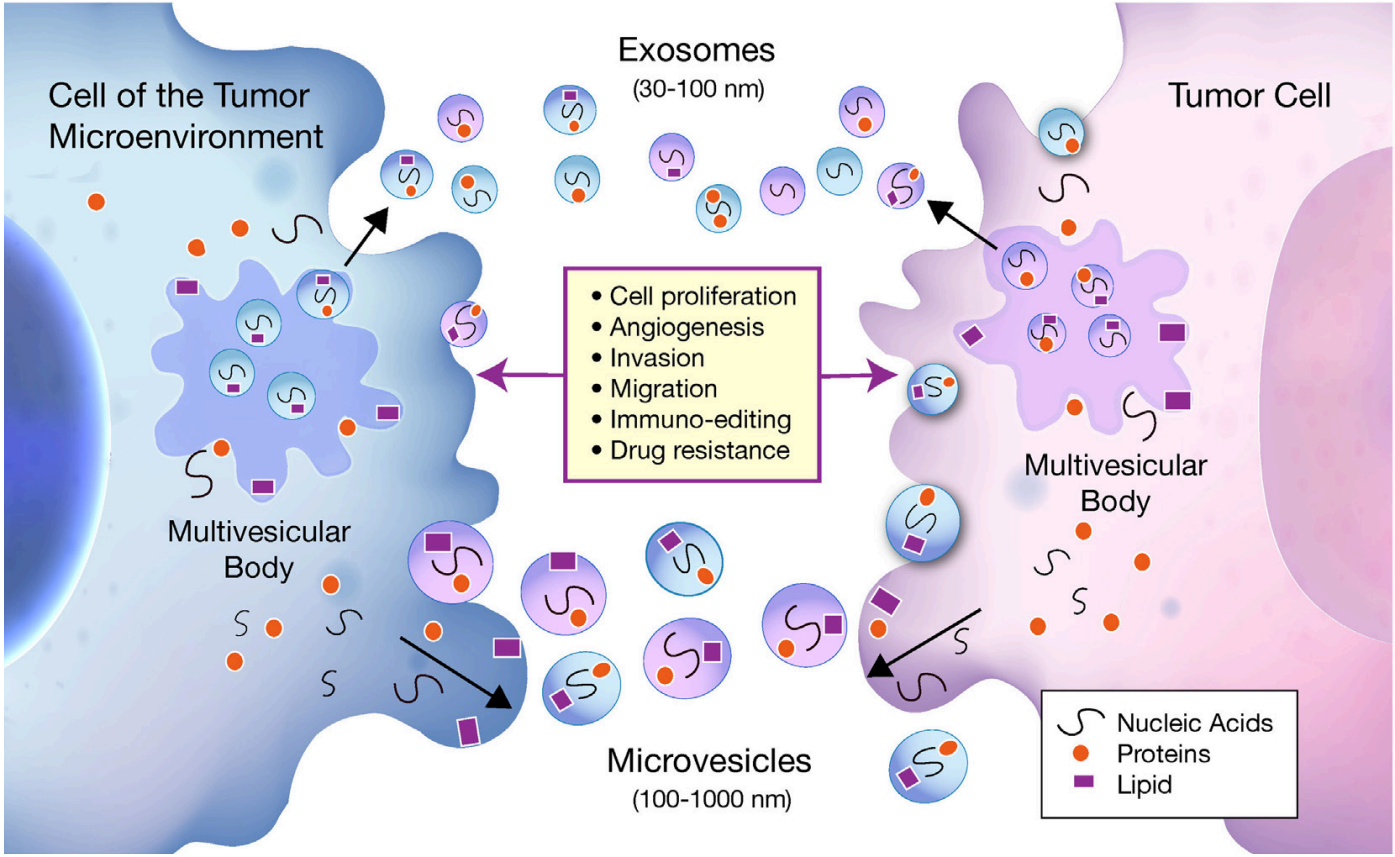
Bidirectional communication occurs between tumor cells and cells of the tumor microenvironment (TME) *via* exosomes and microvesicles (MVs). Exosomes and MVs carry nucleic acids, proteins, and lipids between tumor cells and cells of the TME, which influence a multitude of pathways involved in tumor progression. Exosomes are 30–100 nm in diameter and are generated within larger intracellular multivesicular bodies. They are released into the extracellular environment on fusion with the plasma membrane. MVs generally range from 100 to 1000 nm and are formed when cell components travel to the plasma membrane to be released by membrane budding.

As shown in Figure 1, EVs are classified into two groups depending on their size, biogenesis, and method of release from the cell. Exosomes are 30–100 nm in diameter and are generated within large intracellular multivesicular bodies (35). They are released into the extracellular environment upon fusion with the plasma membrane.

Microvesicles (MVs) generally range from 100 to 1,000 nm and are formed when cell components travel to the plasma membrane to be released by membrane budding (36). Due to an incomplete understanding of exosome and MV biogenesis, and inconsistent methods of purification, the two terms are sometimes used interchangeably within the literature. The classical protocols for purification such as ultracentrifugation, density gradient centrifugation, and newer commercially available kits have been shown to co-isolate MVs and exosomes (37) as well as protein aggregates and other non-EV biomolecules that may interfere with EV specificity (38). Since current isolation methods are not yet standardized, it becomes difficult to assign specific functions to exosomes or MVs independently and why they are both included under the broad classification of EVs.

Extracellular vesicles exhibit wide-ranging roles in maintaining normal cellular and biological physiology. The EV lipid membrane protects its contents from enzyme degradation in bodily fluids making them ideal vessels to send material over a distance. They have been widely studied for their role in immune surveillance, blood coagulation, stem cell maintenance, tissue repair, and development (39). Since EVs play such a pivotal role in maintaining normal cell physiology, it is not surprising that their malfunctioning may lead to disease. EV involvement has been implicated in numerous pathological processes such as CCR5 receptor transport in HIV (40), beta amyloid transmission in Alzheimer’s disease (41), and the spread of prion disease to neighboring cells (42). Undoubtedly, however, the most heavily researched area of EV-associated pathology is their role in tumor development and chemotherapeutic resistance in cancer.

## The Pleotropic Roles of EVs in Cancer Biology

Extracellular vesicles represent a common method of communication within the local TME and distant sites. Although research pertaining to EVs derived from tumor cells monopolizes the literature, more evidence is emerging of the significant roles of stromal cell-derived EVs in cancer biology. EVs isolated from both cell types have been implicated in various steps of tumor growth and development including cell proliferation, angiogenesis, migration, invasion, metastasis, immunoediting, and drug resistance. For a comprehensive list of the molecules involved in tumorigenesis carried by EVs from both cancer and non-cancer cells, which are mentioned in this article, see Table 1.

**Table 1:** Molecules demonstrated to be transported by extracellular vesicles between cancer and non-cancerous cells.

Category of molecule	Cancer cell derived	Non-cancer cell derived
Proliferation	EGFRvIII (43, 44) HSP70, HSP90, Survivin (45, 46) Annexin A6+ (47)	miRNA-21 (48)
Angiogenesis	Vascular endothelial growth factor, FGF2, platelet-derived growth factor (49) Centromere protein E, PDZ-binding kinase, cyclin-dependent kinase 8 (50) miRNA-9 (49)	
Epithelial–mesenchymal transition	Tumor necrosis factor-α, interleukin 6, Akt, β-catenin (51)
Migration/invasion/metastasis	Transforming growth factor (TGF)-β (52) MT1-metalloproteinase (53) Fibronectin (54) miR-181c (55) Integrin alpha-V beta-5, integrin alpha-6 beta-4, integrin alpha-6 beta-1 (56) Migration inhibitory factor (57)	CD81 (58) A disintegrin and metalloproteinase domain-containing protein 10 (59)
Immunoediting	TGF-β (60, 61) Fas ligand (50, 62–64) Galectin 9 (65)	MHC class II molecules (66)
Therapy resistance	P-glycoprotein, MRP1, ABCG2, ABCA3 (67–70)	

### Proliferation

Some of the first evidence of the involvement of EVs in proliferation was in glioblastoma (GBM) cell lines. U373 glioma cells that expressed a highly oncogenic form of epidermal growth factor receptor (EGFR), called EGFRvIII, had the ability to transmit this protein to non-EGFRvIII expressing cells *via* EVs. The recipient cell later expressed the mutated receptor leading to increased rates of proliferation (43). The study further confirmed that the cells expressing the malignant form of EGFR also had increased their rate of EV production to levels easily detected in the blood of its murine host. Later, additional studies also demonstrated the presence EGFRvIII mRNA within the vesicles (44), illustrating the diversity of EV cargo.

Tumor-derived EVs can also interact with and influence cells of the surrounding stroma. Breast cancer-derived EVs carry oncogenic proteins to surrounding fibroblasts inducing transformation and the acquisition of malignant features such as enhanced proliferation and survival (71). In other cancers, heat shock proteins (HSP70 and HSP90) and survivin, which are known to inhibit apoptosis and increase cellular proliferation, have been isolated from tumor-derived EVs and shown to promote a more aggressive cancer phenotype (45, 46).

Some of the important cells of the TME mediate their effect on tumor cells using EVs. For example, CAF-derived EVs containing increased levels of miRNA-21 suppress apoptosis and profoundly promote tumor growth in ovarian cancer (48). Also, increased pancreatic cancer aggressiveness was shown to be dependent on tumor cell-mediated uptake of CAF-derived Annexin A6+ (ANXA6) EVs, and while depletion of ANXA6 in CAFs impaired pancreatic tumor and metastasis occurrence, injection of CAF-derived ANXA6+ EVs enhanced tumorigenesis (47). Human mesenchymal stem cells were also demonstrated to release EVs that lead to the phosphorylation of protein kinase B in gastric cancer cell lines and increased cancer cell proliferation (72).

### Angiogenesis

As a tumor begins to proliferate, it requires increasing amounts of oxygen and nutrients to support the enlarging cellular population, making angiogenesis a necessary mechanism for tumor survival (73). EVs have been implicated in many of the sophisticated processes that allow angiogenesis to occur. In many cancer types, they have been specifically demonstrated to carry proangiogenic factors as cargo including VEGF, FGF2, and PDGF (49). In fact, serum starvation and hypoxic conditions lead to a global increase in EV release from cancer cells (74).

Glioblastoma is characterized by severe hypoxia compared to many other cancer types (75), which results in large areas of necrosis. GBM cells have been demonstrated not only to secrete EVs containing tissue factor, a protein well known in the clotting cascade, but also shown to activate hypoxic endothelial cells leading to the formation of new blood vessels (76). Later studies of GBM also observed that hypoxic tumor-derived EVs significantly enhance angiogenesis both *ex vivo* and *in vitro* by stimulating endothelial cells to secrete soluble factors that trigger P13/AKT signaling in pericytes (77).

RNAs carried by EVs have also been implicated in neo-angiogenic processes. In colorectal cancer, tumor-derived EVs containing cell cycle M-phase-related mRNAs including centromere protein E, PDZ-binding kinase, and cyclin-dependent kinase 8 were demonstrated to promote endothelial proliferation (50). Furthermore, colorectal cancer cell-derived EVs have also been shown to carry miRNAs, including miRNA-9, which causes strong angiogenic effects by suppressing cytokine signaling 5 leading to enhanced activation of Janus kinase and thus driving endothelial cell migration (49).

### Cell Migration, Invasion, and Metastasis

Cancer metastasis is a critical area of cancer biology as it leads to over 90% of solid tumor-related deaths (78). This complex mechanism involves multiple steps including invasion, intravasation into adjacent blood and lymph circulations, evasion of the host immune system, extravasation to distant organs, colonization, and finally the formation of micro- and macro-metastases. EV involvement in the metastatic process has been shown to be extensive as they orchestrate many of these pathological mechanisms.

As mentioned earlier, the CAF is a cell of the tumor stroma that is known to aid in the metastatic process through mechanisms inducing the activation of the EMT. EVs are utilized by tumor cells to help promote the transformation of fibroblasts to CAFs. Tumor-derived EVs from mesothelioma, prostate, bladder, and colorectal cancer cell lines containing TGF-β triggered fibroblast differentiation into a CAF phenotype demonstrated by the expression of alpha-smooth muscle actin (52). Once transformed, CAFs may then also use EVs as a tool to encourage tumor cell migration and metastasis (59). Inducers of the EMT such as TGF-β, tumor necrosis factor alpha (TNF-α), interleukin 6, Akt, and β-catenin are also known cargo of tumor-derived EVs (51). Also, in orthotopic mouse models of breast cancer, EVs containing CD81 were released from CAFs and endocytosed by tumor cells, reloaded with the Wnt11 signaling factor, and re-released to the microenvironment. They then traveled to neighboring cancer cells causing cell polarization and directional motility (58). Another study reported that dermal fibroblasts expressing a CAF phenotype released EVs enriched in A disintegrin and metalloproteinase domain-containing protein 10, which can enhance breast cancer cell motility by activating RhoA and Notch signaling (59).

Extracellular matrix (ECM) remodeling is another important step for tumor invasion and subsequent metastasis to occur. EVs were noted to assist with ECM degradation through transport of metalloproteinases (MMPs) into the extracellular space giving room for cell migration to occur (53). It has also been demonstrated that fibrosarcoma- and melanoma-derived EVs containing MT1-MMP actively degraded type 1 collagen and gelatin (53). After extracellular space is created, tumor-derived EVs carrying ECM molecules such as fibronectin promote cell adhesion and assembly to influence cell directional motility (54).

Tominaga et al. found that the microRNA miR-181c was significantly upregulated in brain metastatic breast cancer cell-derived EVs. When tested in a blood–brain barrier model, miR-181c significantly downregulated the value of the transendothelial electrical resistance (55). They showed that EVs from brain metastatic cancer cells induce the abnormal localization of the tight junction proteins by transferring miR-181c into endothelial cells, which results in the destruction of cell–cell contact. This demonstrated that breast cancer-derived EVs can trigger the breakdown of the blood–brain barrier leading to brain metastasis.

In the 1800s, Paget noticed that different tumor types tend to metastasize to specific organs leading to the “seed and soil” hypothesis of cancer metastasis (79). It is now well established that primary tumors can release cytokines, chemokines, and their receptors to direct metastatic cells to a preferred secondary site called the PMN (10). More recently discovered is that communication between the primary tumor and the PMN can be mediated through EVs. For example, a repertoire of integrins have been reported to guide the vesicles to specific organs. EVs expressing integrin alpha-V beta-5 specifically bind to Kupffer cells mediating liver metastasis, whereas integrin alpha-6 beta-4 and integrin alpha-6 beta-1 bind lung-resident fibroblasts and epithelial cells to mediate lung metastasis (56). Once EVs arrive at the predetermined distant site, their cargo is unloaded to aid in a stepwise creation of the PMN. For example, pancreatic cancer cell EVs were shown to travel to Kupffer cells in the liver to deliver macrophage migration inhibitory factor. This induced the secretion of TGF-β in Kupffer cells and ultimately induced the recruitment of bone marrow-derived cells to complete PMN formation (57). Another important aspect of PMN formation is vascular leakiness, which facilitates the extravasation of malignant cells (80) *via* the delivery of specific molecules that trigger vessel permeabilization of endothelial cells (81) including those carried in EVs. In one study, human breast cancer-derived EVs promoted vascular leakiness in the lung by upregulating S100 proteins and activating Src kinase signaling (56).

### Immunoediting

For tumor cells to progress and survive, they must evade the immune system. In the 1990s, it became established that EVs were used in normal immune cell physiological processes after studies illustrated that B-cells released EVs carrying MHC class-II, co-stimulatory, and adhesion molecules that participate in the immune response (66). Later, studies began to elucidate the complex and dual roles of tumor-derived EVs as both immune activating and suppressing agents and therefore possessing antitumor and protumor properties (82).

Tumor-derived EVs contain specific tumor antigens that may be enriched compared to the donor tumor cell. These antigens can be used as a source to create an enhanced immune response and lead to antitumor activities (83). In fact, this principle has been utilized in therapeutic strategies where dendritic cells from patients with malignant gliomas were exposed to tumor-derived EVs causing the induction of CD8^+^ T-cell-dependent antitumor effects (84). This idea of tumor- or immune cell-derived EVs being used as a cancer vaccine has led to multiple clinical trials (85–88).

Tumor-derived EVs, however, are also involved in multiple methods of immune suppression (89). Numerous studies have demonstrated that immunosuppressive regulatory T-cells (Tregs) can be generated, expanded, and activated by tumor-derived EVs (89). Mesothelioma-derived EVs with TGF-β on the vesicle surface inhibited the proliferative response of CD8^+^ T-cells to interleukin-2 by increasing the number of Tregs (90). Likewise, EVs from colorectal cancer delivered TGF-β to T-cells, which activated the Smad signaling pathway and changed their phenotype to that more similar to Treg cells (91). TGF-β can also directly downregulate cells of the immune system. EVs isolated from acute myeloid leukemia and breast cancer cells were shown to suppress NK cells and T-cells, respectively, by delivering TGF-β directly to the immune cell (60, 61).

Tumor-released miRNAs have more recently been studied as EV cargo causing immune suppression. While surrounded by the EV lipid bilayer, miRNAs are protected from degradation by RNase in the extracellular environment (92). One group studied contents of EVs released from nasopharyngeal carcinoma cells and reported five overexpressed miRNAs that, through inhibition of the MAP-kinase pathway, caused decreased proliferation in T-cells (93). Another group analyzed miRNA levels in dendritic cells that were treated with EVs from pancreatic cancer cells and found increased levels of 9 miRNAs that caused the downregulation of 200 mRNAs and a subsequent decrease in dendritic cell MHC class II expression (94). Furthermore, EV miRNA-cargo also suppresses immune cells by inducing CD4^+^ T-cells to express a more Treg-like phenotype. This activity has been reported in multiple human cancer and mouse tumor models through the reduction of phosphatase and tensin homolog expression (95).

Fas ligand is a well-known protein of the TNF family that induces cell apoptosis on binding to its receptor. Multiple tumor types utilize Fas ligand to induce T-cell death as a means of immune escape (89). Some specific cancers such as melanoma, prostate, oral cancer, and colorectal cancer have demonstrated this ability by specifically releasing EVs containing Fas ligand directly to T-cells (50, 62–64). Galectin-9, like Fas ligand, also mediates apoptosis when bound to its receptor. In nasopharyngeal carcinoma, galactin-9 was delivered to its receptor, Tim-3, on CD4^+^ Th1 helper cells in EVs causing cell death (65).

### Therapy Resistance

The development of chemoradiation and targeted therapy resistance in cancer has remained a challenging hurdle to overcome for cancer treatment. Mechanisms including induction of salvage pathways, drug metabolism alterations, induction of the EMT, and enhanced DNA repair have all been implicated in resistance (96). Significant evidence is emerging that EVs may help facilitate a drug-resistant phenotype through two major methods. First, EVs can help deliver drug efflux pumps (DEPs) or other resistance-acquiring products from drug-resistance tumor cells to drug-sensitive cells (97). Second, within the EV donor cells, DEPs can integrate into the EV itself and help sequester chemotherapeutic drugs to be released into the extracellular environment and leave the donor cell with sublethal concentrations (98).

Drug efflux pumps have long been acknowledged as a major contributor to multidrug resistance (MDR) in cancer, but their associations with EVs have only been recently explored. P-glycoprotein (P-gp) is one of the most well-studied DEPs. The first indication that P-gp was in the cargo of tumor-derived EVs was in leukemia cells. Drug-sensitive tumor cells were co-cultured with EVs isolated from drug-resistant cells, and after 4 h of incubation, the sensitive cells acquired a drug-resistant phenotype and were expressing functional P-gp (67). Other DEPs including MRP1, ABCG2, and ABCA3 are also transferred from resistant to susceptible cells by EVs leading to a MDR phenotype (68–70). Drug-resistant tumor cells can also transfer various other materials to susceptible cells using EVs. A multitude of RNAs including miRNAs, long non-coding RNAs, and functional mRNAs carried by EVs induced a MDR phenotype in previously drug-sensitive cells (99–101). Furthermore, irradiation of a variety of cancer cell lines was noted to cause increased absorption of EVs containing the protein survivin. These cancer cells experienced increased protection from toxic stressors and displayed increased proliferation and metastatic potential (102).

Extracellular vesicles can also be utilized by EV donor cells to aid in drug resistance. Previous studies have illustrated that decreases in pH may cause an increase in EV release from tumor cells (103). Some groups have proposed that within the acidic TME, alkaline drugs may be drawn into the acidic organelles of cancer cells and then eliminated from the cell by EV release (104–106). In fact, it has been shown that EVs released from melanoma cells had cisplatin concentrations that negatively correlated with pH. Furthermore, by using a human xenograft model, they found that treatment with a proton pump inhibitor, which raised the pH of the TME, lead to a decreased level of tumor-derived EV release and EVs containing decreased concentrations of cisplatin (107).

## The Diagnostic Value of EVs

The early detection of cancer is perhaps one of the greatest reasons for a favorable survival (108). Therefore, it is important to investigate novel tools for earlier cancer identification. Since the contents of EVs reflect the cell from which they originate, detection of specific EVs in bodily fluids may have diagnostic potential (109). EVs have been successfully isolated from blood, urine, ascites, cerebro-spinal fluid, amniotic fluid, semen, saliva, and bile (8), and many display a cancer-specific signature that allows for easy detection. For example, EVs isolated from the ascites of colorectal cancer patients contain enriched levels of caludin-3 protein compared to non-pathologic samples (110). Patients with esophageal SCC have serum-isolated EVs enriched with miRNA-21 (111). The specific EV contents also represent a snapshot of the tumor cell status at the time of release. Therefore, they may help in the development of personalized therapeutics or even monitor the tumor’s response to chemotherapy.

Ovarian cancer is known to present with vague and non-specific symptoms. This unfortunate circumstance accounts for the majority of women becoming diagnosed with advanced disease and is why the average 5-year survival is less than 50% (3). Therefore, screening techniques that identify specific cancer biomarkers may be a very useful tool for early detection and a subsequent drop in mortality. One group demonstrated that higher concentrations of an array of miRNAs including miR-21 and miR-141 within EVs were also present in ovarian tumor tissue. Furthermore, higher levels of these miRNAs were associated with more advanced disease (112). Since then, studies have identified other ovarian cancer biomarkers including epithelial cell adhesion molecule and CD24 (113). Efforts are now being made to find practical and cost-effective methods for detecting cancer-specific EVs in patient blood or other bodily fluids.

Scalpel-free biopsies, also known as liquid biopsies, are novel methods for cancer detection. These techniques detect free-floating cancer cells, circulating tumor DNA, or EVs within bodily fluids. There have been varying antibody-based methodologies tested for analyzing EV proteins from bodily fluids without an EV isolation step, including flow cytometry, EV protein microarray, diagnostic magnetic resonance, and nanoplasmonic sensing technology (114). Although still far from standard practice, some companies have already produced commercial kits for detecting lung and prostate cancer where specific EVs are isolated and analyzed using chromatin immunoprecipitation assays (115). In colorectal cancer, an assay called “ExoScreen” can detect cancer-specific circulating double-positive (CD146/CD9) EVs using photosensitive-beads from only 5 µl of patient serum (116).

## EV-Centered Therapy

Given the growing evidence of EV involvement in cancer pathogenesis, it seems intuitive to explore translational approaches that lead to their inhibition. Current studies are utilizing different techniques to inhibit vesicle formation, release, and cell uptake as well as blocking specific components of the EV. The drug amiloride has been shown *in vivo* to block secretion of tumor-derived EVs that contain membrane-associated heat shock protein 72 (HSP72) (117). HSP72 is constitutively expressed in many cancers and is associated with a poor prognosis (118). Furthermore, amiloride was shown to inhibit ceramide, an important mediator of EV biogenesis. Another drug, diannexin, inhibits phosphatidylserine, a regulator of cell adhesion, and EV endocytosis (119). Rab27 is a protein demonstrated to have a significant role in EV secretion (120). In highly metastatic mouse models of both melanoma and breast cancer, knocking down Rab27 led to a significant reduction of tumor EV production, primary tumor size, and metastasis (121, 122). However, since EVs are also essential participants in normal cell physiology, better techniques are required to distinguish and target pathological versus physiological EVs.

As mentioned previously, EVs have been used as cancer vaccines by carrying and providing tumor-specific antigens to immune cells, which prime the immune system and create a powerful immunological response against the tumor. Some of the first phase I clinical trials applying this methodology took place in melanoma and non-small-cell lung carcinoma where dendritic cell-derived EVs were loaded with MHC/tumor antigen and delivered back to patients (86, 87). These studies demonstrated that cancer vaccines are both feasible in creation and safe for administration. Later, EVs from the malignant ascites of colorectal cancer patients were isolated, mixed with specific cytokines, and administered back to the patient as a subcutaneous immunization. The investigators reported that combining ascites EVs with granulocyte-macrophage colony-stimulating factor induced specific antitumor cytotoxic T-lymphocyte activation (85). Furthermore, in a phase II clinical trial including patients with advanced small-cell-lung carcinoma, the administration of dendritic cell-derived EVs caused an increase in natural killer cell activity and longer progression-free survival for patients with low initial expression of natural cytotoxicity receptor NKp30 (88).

The realization that EVs are efficient vehicles for cell-to-cell communication has subsequently given rise to investigations of their use as a method for drug delivery. EVs display many potential advantages over current approaches. They are stable in serum, have specific cell-targeting capabilities, can overcome natural barriers such as the immune system or the blood–brain barrier, and can deliver molecules such as miRNAs or siRNAs that are readily degraded in the serum (38). Multiple methods have been reported to successfully load EVs with a desired drug. Hydrophobic drugs have been demonstrated to integrate with EVs successfully by simply mixing and allowing the drug to pass through the EV lipid bilayer membrane (123). The loading of hydrophilic drugs has proven to be more challenging, but still possible by methods including electroporation, sonication, saponin-mediated permeabilization, and freeze–thaw cycles (124). Perhaps the most challenging aspect of EV-mediated drug deliver is the efficient targeting of specific cell types. Some groups have used transfection-based approaches to encourage cells to express organ-specific ligands or receptors that are loaded into EVs, released from the cell, and then isolated and collected for successive drug loading (124–126). Other groups are experimenting with iron oxide nanoparticles in combination with a drug within EVs to target specific areas of the body by the application of a magnetic field gradient (127).

## Conclusion

Currently, there is a rich source of data that creates a powerful case for the involvement of EVs in most, if not all, aspects of tumor development and progression. The intimate association of EVs with cancer has exciting implications for both cancer diagnostics and therapeutics. However, our general knowledge of EVs and EV-mediated processes remains in its infancy compared to other fields of cancer biology. Perhaps the most important hurdle that is restraining progress is the inability to consistently isolate EV subtypes, preventing investigators from comprehensively comparing and assigning to them specific functions. EV subtype uncertainty is demonstrated in much of the literature when investigators use the broad term “extracellular vesicle” as opposed to “microvesicle” or “exosome.” Isolation techniques cannot just focus on a single characteristic such as size or protein marker to discriminate among EV subtypes. Methods must use a combination of distinguishing features to better enrich the subpopulation, help define specific functions, and later to better categorize their role for therapeutics. Also, since EVs are key players in normal cell physiology, steps need to be taken to avoid interfering with normal processes. Despite current issues with EV isolation and subtyping, their role in cancer progression is dramatic and promises to become crucial in future advances in cancer diagnostics and therapeutics.

## Author Contributions

RS, GM, JH, and LL made substantial contributions to conception and design; RS performed literature search, analyzed data, and wrote the manuscript; GM, XZ, CS, JH, DM, and LL contributed to critically revise the manuscript; JH and LL supervised the study and final approval of the version to be submitted.

## Conflict of Interest Statement

The authors declare that the research was conducted in the absence of any commercial or financial relationships that could be construed as a potential conflict of interest. The reviewer, EP, and handling editor declared their shared affiliation, and the handling editor states that the process nevertheless met the standards of a fair and objective review.
